# Supplementary material for: Content-rich biological network constructed by mining PubMed abstracts
Source: BMC Bioinformatics. 2004 Oct 8;5:147. doi: 10.1186/1471-2105-5-147 (PMC528731; doi:10.1186/1471-2105-5-147)
Supplement: Additional File 2 — The original results of the above study (non-essential files are deleted to keep the file size under the limit set by BMC bioinformatics). [file 1471-2105-5-147-S2.bz2 › chilibotAdditionalFile2/dip10/359ID9195914E5745/html/IL8_IL8RA.html]

 


 **IL8** and **IL8RA** 
  
Found 181 abstracts in PubMed, retrieved 10.  
 

 What does Google say? 
 PDF only 
| .edu only 

---

**Interactive relationship** (e.g. stimulation, inhibition, etc)

**Stimulatory relationship**- When stimulated by tumor necrosis factor alpha, the nonresponsive WM35 melanoma cells synthesize a higher level of CXCR1  [ **IL8RA** ]  and become chemotactic toward interleukin IL 8  [ **IL8** ] .  Ref: 12738812 FASEB J< MedlineTA>FASEB J,
**Neutral relationship**- Interleukin 8  [ **IL8** ]  secreted by endothelial cells induces chemotaxis of melanoma cells through the chemokine receptor CXCR1  [ **IL8RA** ] .  Ref: 12738812 FASEB J< MedlineTA>FASEB J,

**Non-interactive relationship** (e.g. studied together, co-existance, homology, etc.)

- Thus in COPD, in addition to CXCL8  [ **IL8** ]  and CXCR1  [ **IL8RA** ] , CXCL5 and CXCR2 appear to play important roles in the airway neutrophilia characteristic of severe exacerbations.  Ref: 12857718 Am J Respir Crit Care MedAm J Respir Crit Care Med,
- However, the contribution of polymorphisms of the IL 8  [ **IL8** ]  gene and its receptor genes CXCR 1  [ **IL8RA** ]  and CXCR  [ **IL8RA** ]  2 to human TB susceptibility remains untested.  Ref: 12870115 J Infect Dis, 2003
- In this study, we addressed the question of whether expression of the interleukin 8  [ **IL8** ]  receptors IL 8RA CXCR1  [ **IL8RA** ]  and IL 8RB CXCR2 is upregulated in H. pylori infected human gastric biopsy samples.  Ref: 12761119 Infect Immun, 2003
- Both IL 8  [ **IL8** ]  receptors, CXCR1  [ **IL8RA** ]  and CXCR2, are expressed extensively in the fetal intestine.  Ref: 12633567 Cytokine, 2002
- Antibodies against either IL 8  [ **IL8** ]  or CXCR1  [ **IL8RA** ]  inhibit melanoma transendothelial migration in a coculture assay by 30%.  Ref: 12738812 FASEB J< MedlineTA>FASEB J, 2002
- It is well known that chemokines and chemokine receptors, such as interleukin 8  [ **IL8** ]  and its receptors CXCR1  [ **IL8RA** ]  and CXCR2, play important roles in the pathogenesis of psoriasis.  Ref: 12823305 Clin Exp Dermatol, 2003
- By comparison with the NSC and S COPD groups, neutrophilia and gene expression for epithelial derived neutrophil attractant 78 CXCL5, interleukin 8  [ **IL8** ]  CXCL8  [ **IL8** ] , CXCR1  [ **IL8RA** ]  and CXCR2 were each up regulated in the E COPD group p&lt.  Ref: 12857718 Am J Respir Crit Care MedAm J Respir Crit Care Med, 2003
- Expression of the chemokine receptors CCR2, specific for MCP 1, and CXCR1  [ **IL8RA** ]  and CXCR2, specific for IL 8  [ **IL8** ] , were not altered by HCMV infection.  Ref: 12714640 Invest Ophthalmol Vis Sci, 2003
